# Anthocyanins and Their Variation in Red Wines II. Anthocyanin Derived Pigments and Their Color Evolution

**DOI:** 10.3390/molecules17021483

**Published:** 2012-02-07

**Authors:** Fei He, Na-Na Liang, Lin Mu, Qiu-Hong Pan, Jun Wang, Malcolm J. Reeves, Chang-Qing Duan

**Affiliations:** 1 Center for Viticulture and Enology, College of Food Science and Nutritional Engineering, China Agricultural University, Beijing, 100083, China; 2 Faculty of Applied Science, Business and Computing, Eastern Institute of Technology, Napier 4142, New Zealand

**Keywords:** anthocyanin, red wine, pyranoanthocyanin, polymeric anthocyanins, wine maturation and aging

## Abstract

Originating in the grapes, anthocyanins and their derivatives are the crucial pigments responsible for the red wine color. During wine maturation and aging, the concentration of monomeric anthocyanins declines constantly, while numerous more complex and stable anthocyanin derived pigments are formed, mainly including pyranoanthocyanins, polymeric anthocyanins produced from condensation between anthocyanin and/or flavan-3-ols directly or mediated by aldehydes. Correspondingly, their structural modifications result in a characteristic variation of color, from purple-red color in young red wines to brick-red hue of the aged. Because of the extreme complexity of chemical compounds involved, many investigations have been made using model solutions of know composition rather than wine. Thus, there is a large amount of research still required to obtain an overall perspective of the anthocyanin composition and its change with time in red wines. Future findings may well greatly revise our current interpretation of the color in red wines. This paper summarizes the most recent advances in the studies of the anthocyanins derived pigments in red wines, as well as their color evolution.

## 1. Introduction

The color of red wines is one of the first features perceived by consumers that can greatly influence their commercial acceptance [1,2]. Extracted from red grape berries, monomeric anthocyanins contribute crucially to the young red wine color [3,4,5,6]. Meanwhile, the intramolecular or intermolecular interaction of anthocyanins themselves or with other organic chemicals, especially the phenolic compounds, such as sell-association and copigmentation, can further enhance their color expression [7,8,9,10,11,12,13].

During wine maturation and aging, the concentration of monomeric anthocyanins in red wines declines constantly, especially the acylated anthocyanins [2,14,15,16,17]. A series of mechanisms might be related to such changes, such as their adsorption by yeast, their degradation and oxidation, their precipitation with proteins, polysaccharides or condensed tannins, and the progressive and irreversible formation of more complex and stable anthocyanin derived pigments, such as various pyranoanthocyanins, polymeric anthocyanins produced from condensation between anthocyanin and/or flavan-3-ols directly or mediated by aldehydes, as well as their further derivatives [2,14,15,18,19,20,21,22,23,24,25]. Such variations can result in the significant changes of the color, the mouth feel and the flavor properties of red wines [2,4,6,14,15,26,27,28,29].

Until now, numerous mechanisms were proposed to support the formation of such anthocyanin derived pigments, most of which were conducted from studies carried out in model solutions [14,24,25]. However, because the anthocyanin make-up of red wine is extremely complex, as well as other phenolic or non-phenolic compounds, most of the previous studies were conducted in model solutions, which had relatively simple and defined chemical compositions. Although these surveys were under conditions that cannot fully represent the real complexion in red wines, it was still necessary to discover these reactions one by one. As a lot of new research is undertaken, the model wine solutions became to be more complex, which made the studies more difficult [30,31,32,33]. However, they could show us some bigger pieces in the jigsaw puzzle of anthocyanins and their variation in red wines. Furthermore, when future findings in this field give us the intact picture, they might change our current understanding dramatically.

The application of modern analysis methods, especially the solid-phase extraction (SPE) and/or high-performance or high-pressure liquid chromatography (HPLC), greatly facilitate the separation of anthocyanin derivatives in red wines [34,35,36,37]. With the help of various mass spectrometry (MS), such as electrospray ionization mass spectrometry (ESI-MS), matrix-assisted laser desorption/ionization mass spectrometry (MALDI-MS), matrix-assisted laser desorption/ionization time-of-flight mass spectrometry (MALDI-TOF MS), atmospheric pressure photo ionization quadrupole time-of-flight mass spectrometry (APPI-QqTOF MS), as well as the advances in nuclear magnetic resonance (NMR), the anthocyanins derivatives in red wines can be identified in structure quickly and correctly [38,39,40,41,42,43,44]. With the help of such techniques, many of the previous proposed reaction mechanisms for the formation of polymeric anthocyanins and other new pigments have been verified [14,24,25,45,46]. 

The aim of this paper is to summarize both of the basic knowledge and the newest achievements in the field of the formation of anthocyanins derived pigments, their color evolution in aged red wines, and the effects of different aging practices on them.

## 2. Pyranoanthocyanins and Related Pigments in Wines

**Figure 1:**
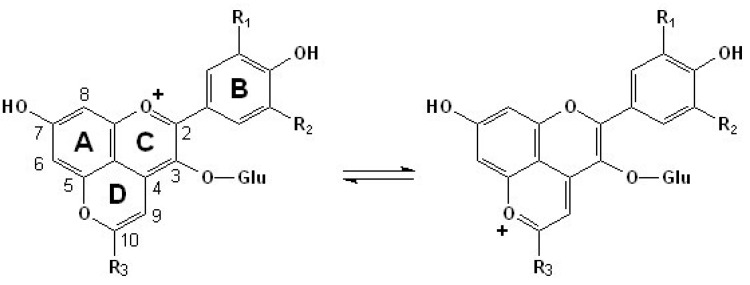
General structures of pyranoanthocyanins derived from anthocyanidin-3-*O*-glucoside in red wines and the dynamic equilibrium between their different flavylium cation forms. The R_1_ and R_2_ groups could be H, OH, OCH_3_. The R_3_ groups could be H, COOH, CH_3_, (vinyl)phenols, (vinyl)flavanols [47,48].

The direct reaction between free anthocyanins and certain yeast by-products, such as acetaldehyde, pyruvic acid and vinylphenols can lead to the formation of another group of stabilized pigments, the pyranoanthocyanins [47,48,49,50,51,52]. Generally, pyranoanthocyanins constitute one of the most important classes of anthocyanin-derived pigments occurring naturally in red wine [53,54,55,56]. Normally, they are cycloaddition products, which have an additional pyran ring between the C4 position in the C ring and the hydroxyl group on the C5 position in the A ring of the anthocyanin molecule [47,52]. More generally speaking, the pyranoanthocyanin structure can be formed through the reaction of an anthocyanin molecule with a compound containing a polarizable double-bound [57]. Thus, compared to the free anthocyanins, pyranoanthocyanins have two heteroaromatic rings, and they have a dynamic equilibrium among different flavylium cation forms, as shown in Figure 1. These new pigments are mainly formed from grape anthocyanins during the fermentation of must and later during the maturation and aging of red wines [47,52].

To date a great number of pyranoanthocyanins have been identified from red wines, especially in the aged red wines, including carboxy-pyranoanthocyanins (A type vitisins), B type vitisins, methyl-pyranoanthocyanins, hydroxyphenyl-pyranoanthocyanins (pinotins), flavanyl-pyranoanthocyanin, and their second generated pigments, such as flavanyl/phenyl-vinylpyranoanthocyanins (portisins), pyranone-anthocyanins (oxovitisins), pyranoanthocyanin dimers and others [25,47]. Very interestingly, some studies even reported that pyranoanthocyanins represented a very big part of total pigment content in rosé wines (partially fermented with skins of red grapes, *V. vinifera* cv. Garnacha) and in blanc de noir (fermented without skins of red grapes, *V. vinifera* cv. Monastrell) base and sparkling wines [58].

Such pyranoanthocyanins are highly stable and resistant to sulfur dioxide bleaching and oxidative degradation, therefore they can significantly contribute to the color stability of red wines [47,52,59]. However, most pyranoanthocyanins possess yellow to orange color and contribute to the tawny color shift associated with red wine aging, except for the newly found A type portisins, which are blue [47,48,50,52,60]. Detailed information of some pyranoanthocyanins that can be frequently detected in aged red wines is summarized in Table 1, as shown below.

**Table 1:** The mass spectral and UV-vis data of some major pyranoanthocyanins that can be detected in various aged red wines [61,62,63,64,65,66,67].

Compounds	Molecular ion M^+^ (*m/z*)	Fragment ion M^+^ ( *m/z*)	λ_max_ (nm)
Vitisin A type			
Cyanidin-3-*O*-glucoside-pyruvic acid	517	359	503
Cyanidin-3-*O*-acetylglucoside-pyruvic acid	559	359	505
Cyanidin-coumaroylglucoside-pyruvic acid	661	359	507
Delphinidin-3-*O*-glucoside-pyruvic acid	533	371	507
Delphinidin-3-*O*-acetylglucoside-pyruvic acid	575	371	509
Delphinidin-3-*O*-coumaroylglucoside-pyruvic acid	679	371	511
Peonidin-3-*O*-glucoside-pyruvic acid	531	369	509
Peonidin-3-*O*-acetylglucoside-pyruvic acid	573	369	510
Peonidin-3-*O*-coumaroylglucoside-pyruvic acid	677	369	511
Petunidin-3-*O*-glucoside-pyruvic acid	547	385	508
Petunidin-3-*O*-acetylglucoside-pyruvic acid	589	385	509
Petunidin-3-*O*-coumaroylglucoside-pyruvic acid	693	385	510
Malvidin-3-*O*-glucoside-pyruvic acid	561	399	513
Malvidin-3-*O*-acetylglucoside-pyruvic acid	603	399	516
Malvidin-3-*O*-coumaroylglucoside-pyruvic acid	707	399	513
Vitisin B type			
Malvidin-3-*O*-glucoside-acetaldehyde	517	355	490
Malvidin-3-*O*-acetylglucoside-acetaldehyde	559	355	494
Malvidin-3-*O*-coumaroylglucoside-acetaldehyde	663	355	497
Pinotin type			
Delphinidin-3-*O*-glucoside-4-vinylcatechol	597	435	510
Delphinidin-3-*O*-acetylglucoside-4-vinylcatechol	639	435	512
Delphinidin-3-*O*-coumaroylglucoside-4-vinylcatechol	743	435	514
Peonidin-3-*O*-glucoside-4-vinylcatechol	595	433	504
Peonidin-3-*O*-acetylglucoside-4-vinylcatechol	637	433	506
Peonidin-3-*O*-coumaroylglucoside-4-vinylcatechol	741	433	508
Petunidin-3-*O*-glucoside-4-vinylcatechol	611	449	510
Petunidin-3-*O*-acetylglucoside-4-vinylcatechol	653	449	512
Petunidin-3-*O*-coumaroylglucoside-4-vinylcatechol	757	449	516
Malvidin-3-*O*-glucoside-4-vinylcatechol	625	463	512
Malvidin-3-*O*-acetylglucoside-4-vinylcatechol	667	463	514
Malvidin-3-*O*-coumaroylglucoside-4-vinylcatechol	771	463	514
Delphinidin-3-*O*-glucoside-4-vinylphenol	581	419	504
Delphinidin-3-*O*-acetylglucoside-4-vinylphenol	623	419	506
Delphinidin-3-*O*-coumaroylglucoside-4-vinylphenol	727	419	506
Peonidin-3-*O*-glucoside-4-vinylphenol	579	417	499
Peonidin-3-*O*-acetylglucoside-4-vinylphenol	621	417	504
Peonidin-3-*O*-coumaroylglucoside-4-vinylphenol	725	417	505
Petunidin-3-*O*-glucoside-4-vinylphenol	595	433	504
Petunidin-3-*O*-acetylglucoside-4-vinylphenol	636	433	506
Petunidin-3-*O*-coumaroylglucoside-4-vinylphenol	741	433	507
Malvidin-3-*O*-glucoside-4-vinylphenol	609	447	504
Malvidin-3-*O*-acetylglucoside-4-vinylphenol	651	447	507
Malvidin-3-*O*-coumaroylglucoside-4-vinylphenol	755	447	509
Malvidin-3-*O*-caffeoylglucoside-4-vinylphenol	771	447	532
Delphinidin-3-*O*-glucoside-4-vinylguaiacol	611	451	502
Peonidin-3-*O*-glucoside-4-vinylguaiacol	609	447	499
Petunidin-3-*O*-glucoside-4-vinylguaiacol	625	463	502
Malvidin-3-*O*-glucoside-4-vinylguaiacol	639	477	504
Malvidin-3-*O*-acetylglucoside-4-vinylguaiacol	681	477	506
Malvidin-3-*O*-coumaroylglucoside-vinylguaiacol	755	477	508
Flavanyl-pyranoanthocyanin type			
Delphinidin-3-*O*-glucoside-4-vinyl(epi)catechin	777	615	501
Delphinidin-3-*O*-acetylglucoside-4-vinyl(epi)catechin	819	615	503
Peonidin-3-*O*-glucoside-4-vinyl(epi)catechin	775	613	199
Peonidin-3-*O*-acetylglucoside-4-vinyl(epi)catechin	817	613	501
Petunidin-3-*O*-glucoside-4-vinyl(epi)catechin	791	629	502
Petunidin-3-*O*-acetylglucoside-4-vinyl(epi)catechin	833	629	504
Malvidin-3-*O*-glucoside-4-vinyl(epi)catechin	805	643	503
Malvidin-3-*O*-acetylglucoside-4-vinyl(epi)catechin	847	643	508
Malvidin-3-*O*-coumaroylglucoside-4-vinyl(epi)catechin	951	643	503

### 2.1. Structures and Formation of Vitisins

**Figure 2:**
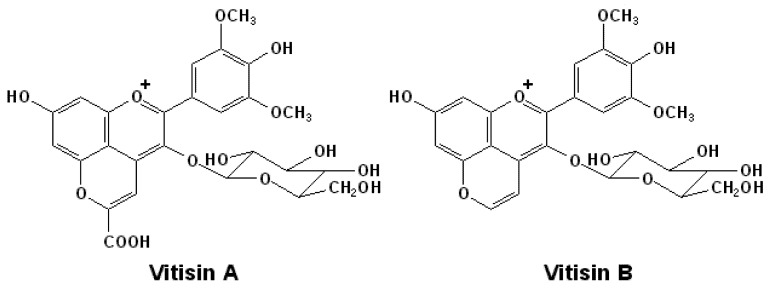
The structures of vitisin A and vitisin B generated from malvidin-3-*O*-glucoside [47,70,71].

Vitisins, the first group of pyranoanthocyanins to be identified in red wines are usually the most abundant pyranoanthocyanins [50,68,69]. The precursors for these pigments are usually secondary metabolites derived from the glycolysis cycle of yeast metabolism during alcoholic fermentation. For example, the most well known, vitisin A, is formed from malvidin-3-*O*-glucoside and pyruvic acid, and vitisin B is synthesized as the product of malvidin-3-*O*-glucoside and acetaldehyde, as shown in Figure 2 [48]. Vitisin A and other similar pyranoanthocyanins arising from reactions between *α*-keto-acids and anthocyanins can also be grouped as carboxy-pyranoanthocyanins, whereas vitisin B or its homologues differ from carboxy-pyranoanthocyanins by lacking the carboxyl group on the D ring of the pyranoanthocyanin molecule [24,70,71]. Other secondary metabolites from yeast, such as acetone, acetoin, oxalacetic acid, acetoacetic acid and diacetyl, are also likely to react with free anthocyanins to form similar pyranoanthocyanins. For example, acetone or acetoacetic acid can react with an anthocyanin by a similar mechanism to form a similar product with a methyl moiety, which can be termed a methyl-pyranoanthocyanin [24,47,60,72].

Besides malvidin-3-*O*-glucoside, other anthocyanin flavylium ions in red wines can also form the vitisin-like pyranoanthocyanins, even the acylated ones. Until now, almost the whole series of such pyranoanthocyanins from anthocyanidin-3-*O*-glucoside have been reported in wine [61,62,63,64]. Structurally, vitisins apparently have a greater conjugation system than their precursors as so should have a longer maximum absorption wavelength than free anthocyanins. However, in reality result is reversed and surprisingly vitisins have a lower maximum absorption wavelength and appear orange under the same pH conditions. The possible reason is that the new formed pyran ring may balance with the B ring contribution resulting in a small decrease in the overall maximum absorption wavelength [73].

**Scheme 1:**
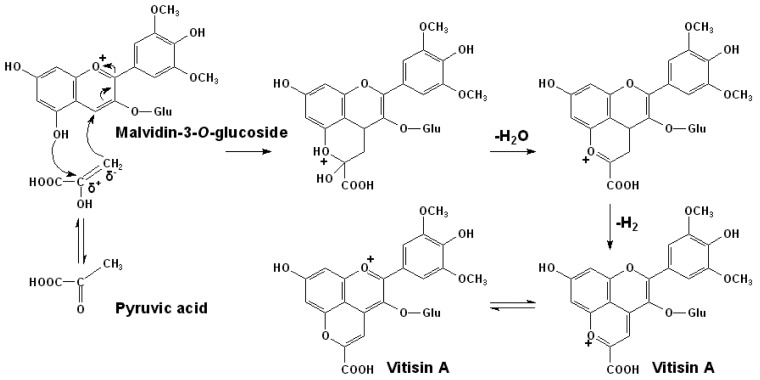
Formation mechanism of vitisin A [47,48,74].

For the formation of vitisin A, under acidic conditions such as exist in red wines, the pyruvic acid in its enolic form can react with malvidin-3-*O*-glucoside at the nucleophilic C5 hydroxyl group and the electrophilic C4 position with the enolic form’s double bond to form the additional pyran ring. This cycloaddition step is followed by further dehydration and re-aromatization to yield the malvidin-3-*O*-glucoside pyruvic adduct, vitisin A, as shown in Scheme 1 [47,48,74].

Normally, vitisins are formed early to middle of the fermentation when the pyruvic acid concentration was more elevated [24,75]. Several factors affect their formation of vitisins. For example, the maximum vitisin A formation was found to occur under conditions of low pH, higher pyruvic acid concentration and low temperature [76,77]. Furthermore, some other factors that can influent the above factors may also affect the production of vitisin A. For example, the yeast strain used in the alcohol fermentation, the lactic acid bacteria used in malolactic fermentation and the content of sulfite dioxide [77,78]. However, other studies also reported that trace amounts of vitisins could be detected and identified in fresh red grape skins or juice, showing the evidence of their existence in grape berries [79,80]. However, the presence of vitisins in grapes may also be artifacts, which should not originate in biosynthesis from grape skins.

Furthermore, vitisins may also participate in the formation of polymers with wine tannins to form more complicated pigments. The existence of (+)-catechin-(C4-C6/C8)-vitisins in red wine has been reported, and it was proposed that they resulted from cycloaddition of pyruvic acid (vitisin A) or acetaldehyde (vitisin B) to the anthocyanin moiety of the (+)-catechin-(C4-C6/C8)-anthocyanins adducts, rather than direct reactions between the nucleophilic C8 or C6 positions of vitisins and the electrophilic position of flavan-3-ols [81].

### 2.2. Structures and Formation of Pinotins

**Figure 3:**
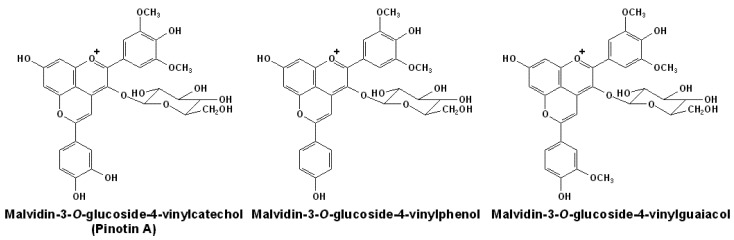
The structures of hydroxyphenyl-pyranoanthocyanins generated from malvidin-3-*O*-glucoside [24,61,82].

In red wines, both hydroxycinnamic acids and 4-vinylphenols can react with free anthocyanins to form the hydroxyphenyl-pyranoanthocyanins, some of which are also named as pinotins, since they were firstly isolated from Pinotage wines [24,47,82,83,84]. The basic structures of well known hydroxyphenyl-pyranoanthocyanins are illustrated in Figure 3. 

In red wines, the main hydroxycinnamic acids present are *p*-coumaric, caffeic, ferulic and sinapic acids, whereas 4-vinylphenol and 4-vinylguaiacol may normally arise from the decarboxylation of *p*-coumaric and ferulic acid, respectively [85]. In a previously proposed mechanism, hydroxyphenyl-pyranoanthocyanins result from the cycloaddition of the ethylenic bond of the 4-vinylphenol molecule at C4 and C5 positions of the anthocyanin molecule followed by an oxidation process, resulting in a pyran ring, and hydroxycinnamic acids cannot be involved in the reactions without the enzymatic decarboxylation via the *Saccharomyces cerevisiae* cinnamate decarboxylase (CD) during alcoholic fermentation [85,86].

**Scheme 2:**
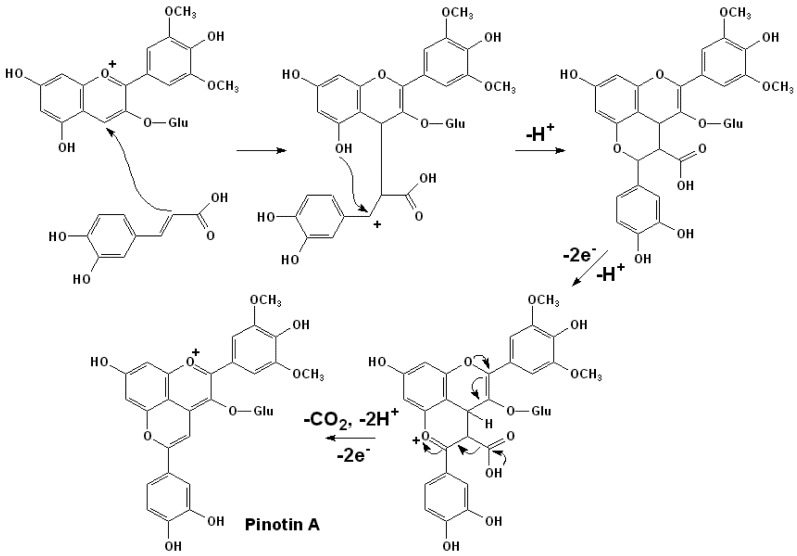
Formation mechanism of Pinotin A [51,87].

However, a relatively new mechanism was reported to result in the formation of such group pyranoanthocyanins. In the formation of pinotin A (pyranomalvidin-3-*O*-glucoside-catechol), the best known pigment in this category, the nucleophilic C2 position of the caffeic acid initially attacks the electrophilic C4 position of a malvidin-3-*O*-glucoside to form an electro deficient intermediate. Subsequently, the hydroxyl group at the C5 position of the anthocyanin moiety will intramolecularly react with this intermediate carbonium ion to form a pyran ring. After the further oxidation and decarboxylation, the final products are formed, as shown in Scheme 2. Thus, without the enzymatic decarboxylation by yeast CD into their respective 4-vinylphenols, but with the additional non-enzymatic decarboxylation, hydroxycinnamic acids with electron-donor substituent, such as *p*-coumaric, ferulic, caffeic and sinapic acids can also participate in the formation of pinotin-like pigments with free anthocyanins [47,51,87].

Until now, almost the whole series of the phenyl-pyranoanthocyanin derivatives of anthocyanidin-3-*O*-glucoside and their acylated derivates are detected and identified in red wines [87,88]. At the acidic pH of red wines, these pigments absorb in the visible spectrum at λ_max_ 505–508 nm, showing it a red-orange color [47,61,62,63,64]. The concentration of hydroxycinnamic acids or 4-vinylphenols is more important than that of the anthocyanidin-3-*O*-glucosides in the formation of such kinds of pyranoanthocyanins [89]. Unlike vitisins, they tend to accumulate post alcohol fermentation. For example, the most rapid synthesis of pinotin A was observed in 2.5 to 4 year old wines where the malvidin-3-*O*-glucoside had decreased to an extremely low level while the concentration of caffeic acid remained very stable. A minimum concentration of 5–10 mg/L of malvidin-3-*O*-glucoside is necessary for such reactions [90].

### 2.3. Structures and Formation of Flavanyl-Pyranoanthocyanins

**Figure 4:**
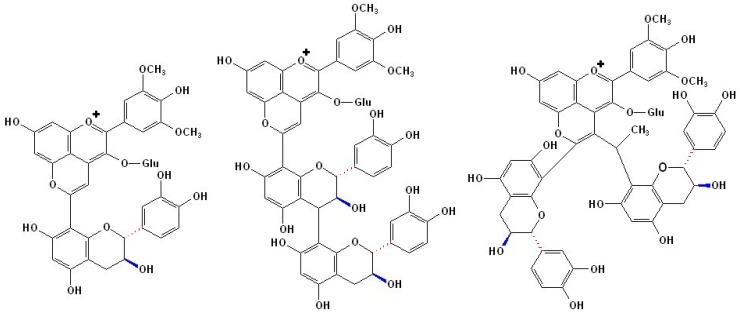
The structures of flavanyl-pyranoanthocyanins generated from malvidin-3-*O*-glucoside and 8-vinylcatechin or 8-vinylprocyanidin [47,92].

Flavanyl-pyranoanthocyanins, also known as vinylﬂavanol-pyranoanthocyanins or pyrano-anthocyanin-flavanols, bearing a pyranoanthocyanin moiety directly linked to flavanols, are another group of pyranoanthocyanins naturally occurring in red wines or synthesized in wine model solutions by reactions of anthocyanins with flavanols mediated by acetaldehyde, as shown in Figure 4 [24,47]. Similar to vitisins or pinotins, these pigments possess a hypsochromically shifted maximum of absorption in the visible region to values of 490–511 nm. Compared to the red-purple hue of the genuine anthocyanins, this hypsochromic shift of λ_max _results in a more orange color, which is also more stable at varying pH values [91].

**Scheme 3:**
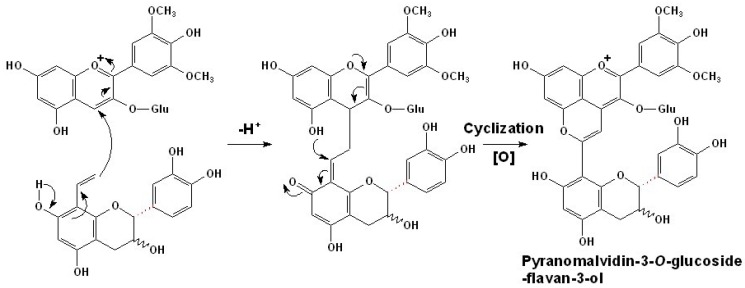
Formation mechanism of flavanyl-pyranoanthocyanins [47,92].

Pigments of such class, such as adducts of malvidin-3-*O*-glucoside and vinylcatechin, vinylepicatechin, and vinylprocyanidin B2, were first detected and identified in model solutions [91]. Later, flavanyl-pyranoanthocyanins derived from malvidin-3-*O*-glucoside (as well as its acylated products) and the vinyl derivatives of (+)-catechin, (−)-epicatechin, and oligomeric procyanidins up to tetramers were detected in red wines by HPLC-MS/MS analysis [93]. It is proposed that they are formed by the cycloaddition reaction between vinylflavanols and anthocyanins, by a mechanism similar to that of hydroxyphenyl-pyranoanthocyanins with vinylphenols, as shown in Scheme 3 [92]. However, as important precursors, vinylflavanol adducts do not occur naturally in grapes. It is believed that vinylflavanol adducts arise from the dehydration of flavanol-ethanol adducts and the decomposition of the methylmethine-linked flavanol adducts, both of which can result from the reaction between flavanols and acetaldehyde [92,93,94,95]. 

Besides pyranoanthocyanin-flavanol monomers, pyranoanthocyanin-procyanidin dimers and pyranoanthocyanin-flavanols with more polymerized structures have also been identified from some Port wines and red wines [63,66,67,93]. Sometimes, malvidin-3-*O*-glucoside can react with 8-vinylcatechin to produce various more complicate pigments, including the tree-like pyranoanthocyanin. However, such products usually cannot be detected in wine samples because of their trace levels [92]. Further, such pyranoanthocyanin-flavanols were much more stable against degradation and bleaching by sulfur dioxide than the free anthocyanins [66].

### 2.4. Structures and Formation of Portisins

Also known as flavanyl/phenyl-vinylpyranoanthocyanins, the portisins were firstly isolated and identified in Port red wines [24,47,96,97]. As a group of significant anthocyanin derivatives, pyranoanthocyanins, especially the carboxy-pyranoanthocyanins, can also react with other compounds during wine aging to generate some other pyranoanthocyanins with more complicated structures. Structurally, they bear a pyranoanthocyanin moiety linked through a vinyl bridge to a flavanol or phenol unit [98]. As may be expected portisins, compared with vitisins, have maximum light absorption at a longer wavelength, close to 570 nm, and therefore present a blue-violet color [96,97,98,99]. Furthermore, portisins seems to have higher resistance to the attack of water or sulfite dioxides than their monomeric anthocyanin precursors, which could be explained by a higher protection of their chromophore groups against water or bisulfite nucleophilic attack [99,100].

**Scheme 4:**
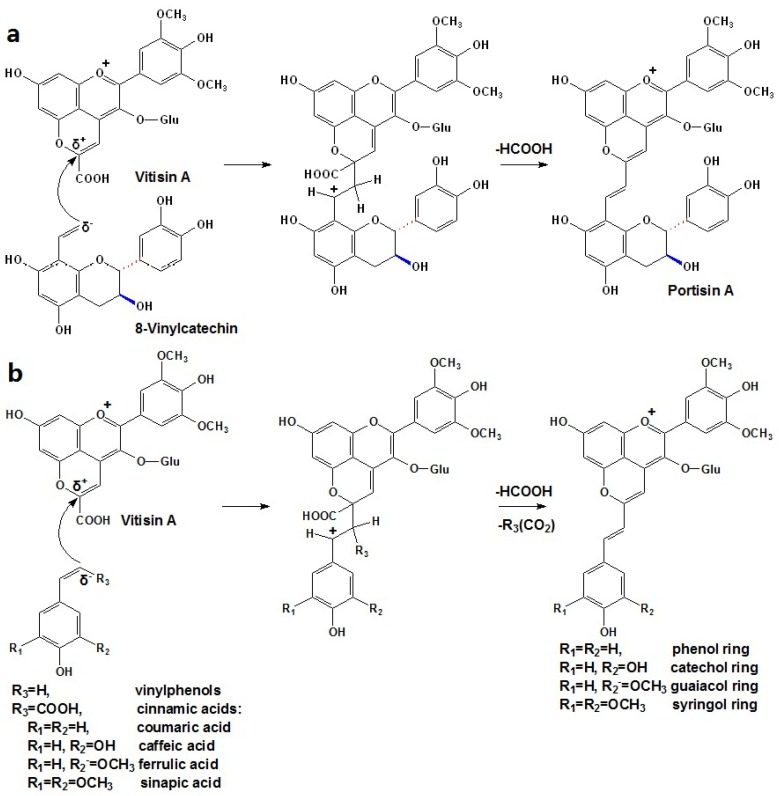
Formation mechanism of flavanyl/phenyl-vinylpyranoanthocyanin derived from carboxy-pyranoanthocyanins: (**a**) A type portisins (flavanyl-vinylpyranoanthocyanin); (**b**) B type portisins (phenyl-vinylpyranoanthocyanin) [96,103].

Portisins are considered to be derived through condensation of anthocyanin-pyruvic acid adducts (vitisin A type pyranoanthocyanins) and vinylphenolic compounds. In the formation of portisin A, a vitisin A molecule reacts through its carbon at the C10 position with the vinyl group of a 8-vinyl-flavanol adduct, which may be derived either from the cleavage of ethyl-linked flavanol oligomers, or from the cleavage of anthocyanin-ethyl-flavanol pigments. After the loss of a formic acid group and further oxidation, a vinyl bridge is formed and a new pigment is the result, as shown in Scheme 4a [96,100,101]. The extended conjugation of the π electrons in the newly formed structure may confer a higher stability of the molecule and may be the origin of its blue color. Although these compounds are in very low concentration in red wines, their possible chromatic contribution should not be overlooked [96,97,98,99]. Until now, many A type portisins has been identified in some Port wine fractions, including the catechin-vinylpyrano derivatives of petunidin, peonidin and malvidin-3-*O*-glucosides, malvidin-3-*O*-acetylglucoside and peonidin, as well as malvidin-3-*O*-coumaroylglucoside [102].

Recently, a new group of portisins, B type, were detected in aged Port wines. In these new pigments, the flavanol moiety is replaced by a phenolic moiety with different hydroxylation and methoxylation patterns [98,103]. Correspondingly, they are proposed to be formed by the reaction of carboxy-pyranoanthocyanins with cinnamic acids or vinylphenols. This mechanism is similar to that for portisin A, but it involves a further decarboxylation, as shown in Figure 8b [103]. However, the colors of B type portisins are different from those of A type portisins, by showing a hypsochromic shift of the maximum absorption wavelength in the visible spectrum [73,103].

### 2.5. Structures and Formation of Oxovitisins

**Scheme 5:**
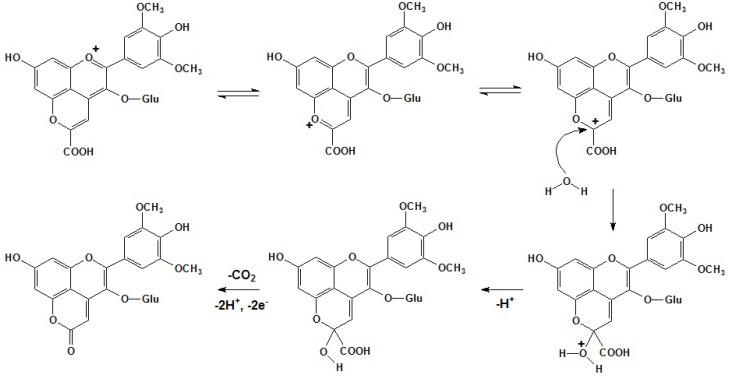
Formation mechanism of oxovitisins (pyranone-anthocyanin) derived from carboxy-pyranoanthocyanins [104].

Besides portisins, carboxy-pyranoanthocyanin, because of their electrophilic character, can also be involved in the formation of other pyranoanthocyanin pigments. More recently, a new class pyranoanthocyanins, named as oxovitisins (pyranone-anthocyanin) was detected in an aged Port wine. These new pigments display only a pronounced broad band around 370 nm in the UV-Vis spectrum and therefore contribute yellow color to a wine. Generally, they are direct derivatives of carboxy-pyranoanthocyanins, especially vitisin A. In their formation, a water molecule firstly attacks the positively charged C10 position of the pyranoanthocyanin precursor, leading to their hemiacetal formation. Then the resulting intermediates undergo a series of decarboxylation, oxidation, and dehydration reactions to produce the final new and neutral pyran-2-one structures, as shown in Scheme 5 [104]. However, it was recently reported that vitisin B types are not in equilibrium with the hemiacetal forms resulting from the nucleophilic attack by water, thus these vitisins may not readily take part in the formation of such a class of pigments [47,104,105].

### 2.6. Pyranoanthocyanin Dimers

**Scheme 6:**
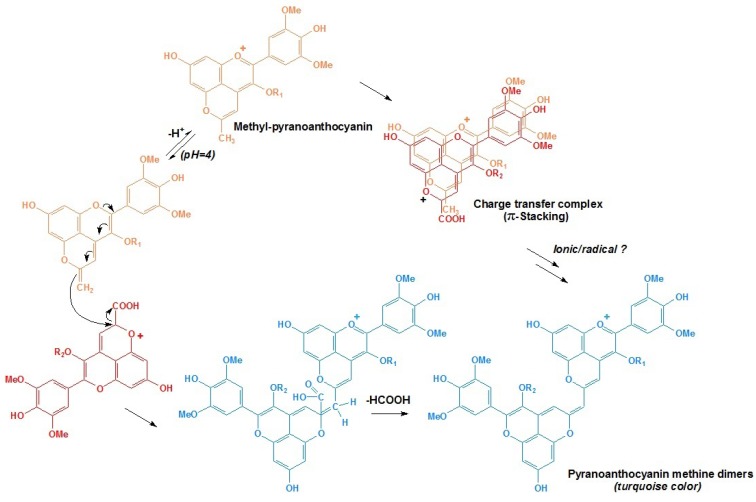
Proposed two pathways for the formation of pyranoanthocyanin dimers [47,106].

More recently, a new class of pyranoanthocyanin dimers that present an outstanding, rare turquoise color (maximum visible absorption wavelength at ~730 and ~680 nm) was identified in an aged Port wine [106]. They were shown to be formed from reactions between carboxy-pyranoanthocyanins and methyl-pyranoanthocyanins. Two mechanisms for their synthesis have been proposed, as shown in Scheme 6. In the first pathway, after the deprotonation of the methyl group of the methyl-pyranoanthocyanin, a methylene group is formed at C10 position of the E ring. Then, the nucleophilic double bond of this methylene group attacks the electrophilic carbon at C10 position of the carboxy-pyranoanthocyanin molecule. After losing formic acid, the pyranoanthocyanin methane dimer with a structure of two pyranoanthocyanin moieties linked through a methine group can be formed. The second pathway involves the formation of a charge-transfer complex between the two precursors which are stabilized by the π-stacking of the aromatic rings. Through further ionic or radical reactions, condensation occurs between both the two precursors. Although there are still some unclear assumptions in the second mechanism, it was proposed that the charge-transfer complex pathway seemed to be more likely [47,106,107].

## 3. Polymeric Anthocyanins

Besides their important role as cofactors in copigmentation, phenolic or polyphenolic compounds extracted from the red grape berry skins and seeds, or even from the oak wood, can also be directly involved in the wine coloration by forming polymeric pigments with anthocyanins [14,24,45,108,109]. The formation of such pigments occurs by direct polymerization of anthocyanins and flavan-3-ols or proanthocyanidins, as well as by the formation of the ‘bridge’ mediated polymeric pigments from anthocyanins themselves or with flavan-3-ols or proanthocyanidins [14,24,45]. 

These polymeric pigments are more stable than the monomeric anthocyanins and help stabilize wine color. Polymerization results in the chromophore of the anthocyanin being protected from water and nucleophilic attack, oxidation or other chemical modifications, such as the bleaching of sulfur dioxide [14,110,111]. In addition, as a red wine ages the proportion of monomeric anthocyanins soon decreases as the colored polymeric anthocyanins form. Consequently this process affects the anthocyanin equilibrium while retaining the red color of anthocyanins in the polymer [112]. Besides, such polymeric pigments are also important to the mouth feel of red wines, since they are more soluble than the pure polymeric proanthocyanidins and limit the precipitation of these condensed tannins. It is estimated that about 25% anthocyanins may have polymerized with flavonoid compounds by the end of alcohol fermentation, and this level will rise to more 40% after one year’s aging. From then on, the polymerization will continue at a decreasing rate until there has been total polymerization after several years [14,113]. It is obvious that after long time, though there are almost no monomeric anthocyanins in old red wines, the wines are still red or red-brown in hue [114]. So with time polymerization shifts the absorption prosperities of anthocyanin chromophore from red to brown, or even orange and yellow [115]. The color density also diminishes with time, as a result of the loss of monomeric anthocyanins and the slow precipitation of large pigment polymers.

**Scheme 7:**
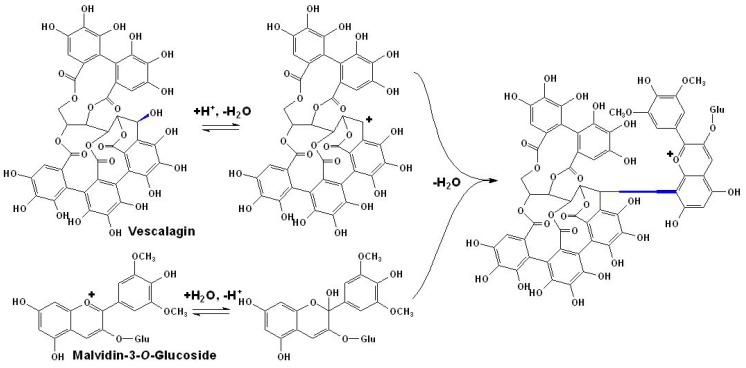
Formation mechanism of ‘anthocyanoellagitannin’ pigment derived from malvidin-3-*O*-glucoside and (−)-vescalagin [109].

It is worth to point that besides flavonoids, anthocyanins may react with some non-flavonoid phenolic compounds to form complicated pigments. In a research conducted in an acidic organic solution, malvidin-3-*O*-glucoside can react with (−)-vescalagin to form desired condensation product. The visible absorption band of such so-called ‘anthocyanoellagitannin’ pigment is bathochromically shifted by more than 20 nm, presenting a deeper red-purple color [109]. Although such anthocyanin derivatives have not been observed in red wines, considering (−)-vescalagin or its epimer (-)-castalagin are found in relatively high amounts in fagaceous hardwoods, such as in *Quercus* (oak) and *Castanea* (chestnut) species (up to 6% by weight of dry heartwood), it is not hard to imagine the detection and identification of pigments of this class in the red wines after wood aging in the near future [109]. The proposed formation mechanism of the ‘anthocyanoellagitannin’ pigment was illustrated in Scheme 7, as shown below [109].

### 3.1. Directly Condensed Products of Anthocyanins and Flavanols

**Scheme 8:**
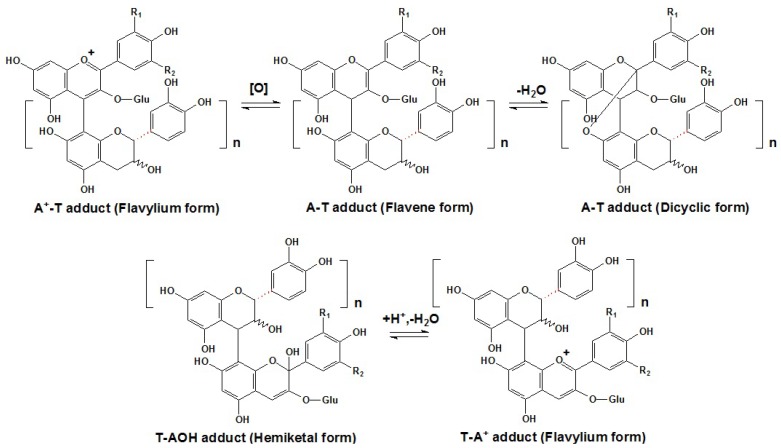
The equilibria among the different structural forms of T-A or A-T type anthocyanin/tannin adducts in aged red wines [113].

At red wine pH, free anthocyanins in the flavylium form can act as electrophiles through their C2 or C4 positions in the C ring, or act as nucleophiles in the hemiketal form through their C6 or C8 positions in the A ring [14,46,116,117,118,119]. Thus, free anthocyanins can condense with tannins (primarily flavan-3-ols and oligomeric proanthocyanidins) directly to generate either T-A or A-T type anthocyanin/tannin adducts [14,46,112,116,118,119,120]. As with monomeric anthocyanins, these polymeric pigments can also occur in a dynamic equilibrium among some molecular forms, mainly the quinoidal base, the flavylium cation and the hemiketal or carbinol pseudobase, as shown in Scheme 8 [116]. However, the presence of oxygen or oxidants is necessary for the color recovery [121,122]. Additionally, acidic pH conditions and high temperatures can also promote such reactions [117,122].

**Scheme 9:**
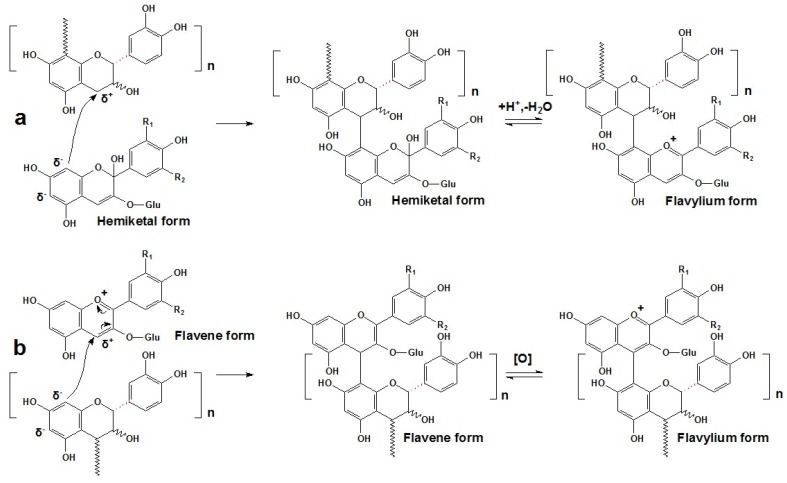
Proposed mechanism of T-A and A-T adducts formation [112,118,119,120,123].

The abundant and colorless anthocyanins in hemiketal forms can generate the T-A adducts with tannins. They are formed by the attack of the nucleophilic C8 or C6 position in the A ring of the anthocyanin molecule to the electrophilic C4 position in the C ring of a flavan-3-ol or a terminal unit of oligomeric proanthocyanidin. After dehydration, the T-A adducts can generate colored flavylium chromophores and enhance the color expression, since the colorless hemiketal forms of free anthocyanins are modified to their corresponding colored flavylium forms of polymeric anthocyanins, as shown in Scheme 9a [14,46,112,118,119,120,123]. The presence of such adducts has been extensively reported in red wines, but their exact structures were only elucidated recently by MS and NMR [112,118,119,124,125]. For example, (+)-catechin-(C4α→C8)-malvidin-3-*O*-glucoside is the most well known polymerized pigment in this category [118,119]. It is reported that the oligomeric proanthocyanidins can possess up to eight flavan-3-ol units, but it is possible that new pigments containing proanthocyanidins with higher polymerization may be discovered [123].

**Figure 5:**
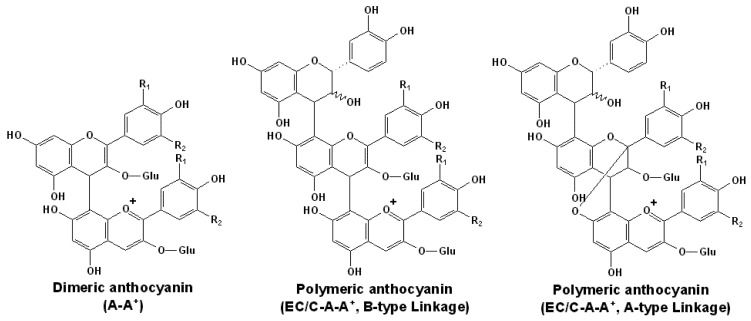
Structures of some directly condensed oligomeric anthocyanins [133,134,135].

In contrast, when the electrophilic C4 position in the C ring of an anthocyanin in the flavylium form is attacked by the nucleophilic C8 or C6 position in the A ring of a flavan-3-ol or a terminal unit of an additional proanthocyanidin, an A-T adduct can be produced. Initially, this adduct is generated as a colorless flavene complex [126]. It is thought that the subsequent oxidation can rearrange the molecule to the colored flavylium state, as shown in Figure 13b [127,128]. Some of them can even form the anthocyanin-flavan-3-ol dimer, which contains both carbon-carbon and ether interflavanoid linkages [129]. Besides, the diglucosidic anthocyanins can also participate in such polymerization [130]. Because the C4 position of the flavylium anthocyanin is utilized in the A-T type adduct, anthocyanins in the bisulfite addition compound form, induced by the addition of sulfur dioxide to the C4 carbon, may prevent such A-T polymerization [131,132]. Additionally, anthocyanin-anthocyanin dimers can also be formed in red wines. Interestingly, some of them have the structures that are similar to B-type proanthocyanidins, but others can form an additional intramolecular bond by an additional dehydration reaction to produce an A-type proanthocyanidin like structure, as shown in Figure 5 [133,134,135,136]. For example, besides the normal malvidin-3-*O*-glucoside-(C4-C8)-malvidin-3-*O*-glucoside, the bicyclic malvidin-3-*O*-glucoside-(C2-*O*-C7, C4-C8)-malvidin-3-*O*-glucoside was also detected in red wine fractions [133,134,135]. Detailed mass spectrometry information of some major products from direct condensation reactions of anthocyanins and/or flavan-3-ols that can be detected in aged red wines or be synthesized in model wine solutions is summarized in Table 2, as shown below [128,134,135].

As well as the above flavan-3-ol monomers and oligomeric proanthocyanidins can also undergo a similar condensation generating larger condensed tannins, which might be more complex than those extracted from grape berries [137]. However, these large polymers are easier to precipitate and harder to condense with anthocyanins than the smaller proanthocyanidins [138].

**Table 2:** The mass spectral data of some major products from direct condensation reactions of anthocyanins and/or flavan-3-ols [128,134,135].

Compounds	Molecular ion M^+^ (*m/z*)	Fragment ion M^+^ (*m/z*)
Delphinidin-glucoside-(epi)catechin	753	591,573,465,439,303
Petunidin-glucoside-(epi)catechin	767	605,587,453,359,329
Peonidin-glucoside-(epi)catechin	751	589,571,463,437
Malvidin-glucoside-gallocatechin	797	635,617,509,467,373
Malvidin-glucoside-(epi)catechin	781	619,601,467,373,331
Malvidin-acetylglucoside-(epi)catechin	823	619,601,467,493,331
Malvidin-coumaroylglucoside-(epi)catechin	927	619,493,467,451,331
Malvidin-glucoside-PC dimer	1069	907,781,619
Malvidin-glucoside-malvidin-glucoside	985	823,661,535,331
Malvidin-glucoside-malvidin-acetylglucoside	1027	865,823,661,331
Malvidin-glucoside-petunidin-acetylglucoside	1013	851,809,647,331
Malvidin-glucoside-delphinidin-acetylglucoside	999	837,795,633,331,303
Malvidin-acetylglucoside-malvidin-acetylglucoside	1069	865,661,331
Malvidin-glucoside-peonidin-acetylglucoside	997	835,631,303
Malvidin-acetylglucoside-petunidin-acetylglucoside	1055	851,647,521,317
Malvidin-glucoside-malvidin-coumaroylglucoside	1131	969,823,661,535,331
Malvidin-acetylglucoside-malvidin-coumaroylglucoside	1173	969,865,661,535,331
Malvidin-glucoside-delphinidin-coumaroylglucoside	1103	941,795,633,507,331
Malvidin-glucoside-petunidin-coumaroylglucoside	1117	955,809,647,317
Malvidin-glucoside-peonidin-coumaroylglucoside	1101	939,793,631,505,331
Malvidin-coumaroylglucoside-malvidin-coumaroylglucoside	1277	969,661,639
Malvidin-glucoside-delphinidin-glucoside	957	795,633,507,331,303
Malvidin-glucoside-cyanidin-glucoside	941	779,617,491,449
Malvidin-glucoside-petunidin-glucoside	971	809,647,521,331,317
Malvidin-glucoside-peonidin-glucoside	955	793,631,505,331
Malvidin-glucoside-cyanidin-coumaroylglucoside	1087	925,779,617,493,287
(Epi)catechin-delphinidin-glucoside-malvidin-glucoside	1245	1083,795,921,903,633
(Epi)catechin-cyanidin-glucoside-malvidin-glucoside	1229	1067,917,904
(Epi)catechin-petunidin-glucoside-malvidin-glucoside	1259	1097,971,935,747,671
(Epi)catechin-peonidin-glucoside-malvidin-glucoside	1243	1081,1063,919
(Epi)catechin-malvidin-glucoside-malvidin-glucoside	1273	1111,949,931,823,661
(Epi)gallocatechin-delphinidin-glucoside-malvidin-glucoside	1261	1099,937
(Epi)gallocatechin-petunidin-glucoside-malvidin-glucoside	1275	1113,951,647
(Epi)gallocatechin-malvidin-glucoside-malvidin-glucoside	1289	1127,965,823,535,331

### 3.2. Acetaldehyde or Glyoxylic Acid Mediated Polymeric Products

**Figure 6:**
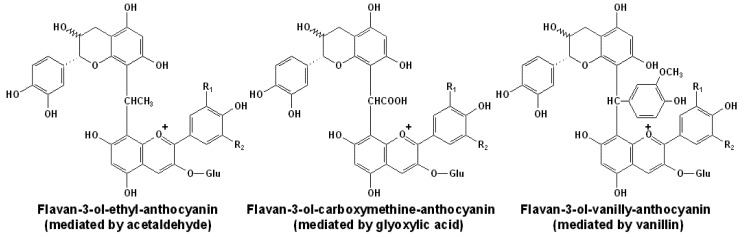
Structures of some mediated condensed oligomeric anthocyanins [91,139,140,141,142].

Besides the direct condensation with flavonoids, anthocyanins can also form reddish or violet polymeric pigments via the mediation of aldehydes, showing in Figure 6 [91,139,140,141,142]. Studies indicated that the anthocyanin moiety in such polymeric pigment was more protected against water attack and the color of such pigment showed more stability with regard to bleaching by sulfite dioxide than that of monomeric anthocyanins. However, they are more sensitive to degradation in aqueous solutions [143]. 

**Scheme 10:**
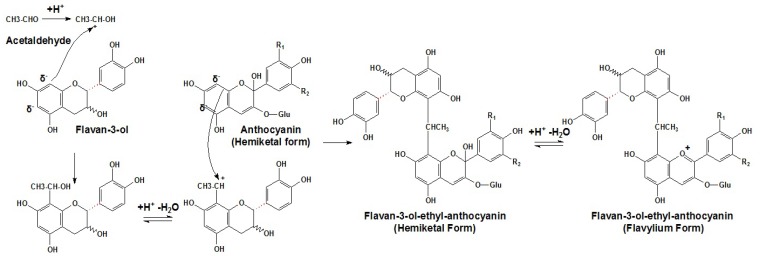
Proposed mechanism of acetaldehyde mediated tannin–anthocyanin additions [32,144,145].

Arising from different sources, such as yeast or the oxidation of ethanol, acetaldehyde is the most abundant aldehyde in red wine [139]. At the low pH value of red wines, a small proportion of acetaldehyde exists as the protonated form, as a reactive carbonium ion state. In such state, acetaldehyde can react with the nucleophilic C8 or C6 position in the A ring of a flavan-3-ol molecule or a terminal unit of proanthocyanidin. After dehydration, the acetaldehyde linked flavanol adduct can give rise to a new carbonium ion that can attack the nucleophilic C8 position in the A ring of an anthocyanin molecule. After deprotonation, the resulting compound can form a violet quinoidal base of the cross-linked flavanol-ethyl-anthocyanin adduct, as shown in Scheme 10 [32,144,145].

As the flavanol moiety can be attached at the C8 or C6 position, whereas the anthocyanin moiety in the flavylium state can be only attached at the C8 position, such indirect polymerized pigments have two different structural isomers, involving the C8-(CH-CH_3_)-CH8 and C6-(CH-CH_3_)-CH8 isomers [24,140,146]. Because the ethyl (or methylmethine) bridge has an asymmetric carbon, each of the above mentioned regioisomers has two diastereoisomers differing in the stereochemistry (*R* or *S*) of the methine carbon [117,140]. This reaction mechanism has been demonstrated in numerous studies using the NMR analysis of indirect condensed products, such as the ethyl-linked malvidin-3-*O*-glucoside-(epi)catechin adducts [147]. However, recently it has also been demonstrated that the C6 position in the A ring of the hemiketal form of an anthocyanin can also participate in such condensation, albeit to a lesser extent than the C8 position [148]. The CH_3_CH linked new pigments can cause rapid and spectacular color augmentation with shifts toward violet [93,141,149].

Additionally, ethyl-linked pigments usually undergo further polycondensation, which means the degree of polymerization can be very high. Previous studies have already identified trimeric and tetrameric pigments in model solutions [95]. It is important to note that anthocyanins are only present at the terminal points of the linear oligomers linked by ethyl bridging, thus there are no ethyl-linked pigments containing more than two anthocyanin units [95,150]. Recently, some more complicated ethyl bridged flavanol-anthocyanin oligomeric adducts, including (+)-catechin-ethyl-(+)-catechin-malvidin-3-*O*-glucoside and ethyl-[(+)-catechin-malvidin-3-*O*-glucoside]_2_, were obtained by hemisynthesis and identified in aged table wine and aged Port wine, showing the possible existence of more complicated condensed anthocyanin pigments [148,150].

The polymerization of monomeric flavan-3-ols and oligomeric proanthocyanidins with anthocyanins in the presence of acetaldehyde occurs at different rates. For example, when malvidin-3-*O*-glucoside participates in the acetaldehyde mediated polymerization, the reaction rates decrease in the following order: procyanidin C1, procyanidin B1, procyanidin B2, procyanidin B2-3′-*O*-galloyl, procyanidin B3, (−)-epicatechin and (+)-catechin [151]. When procyanidin B2 participates in the acetaldehyde mediated polymerization, the reaction rates decrease as the following order: peonidin-3-*O*-glucoside, cyanidin-3-*O*-glucoside and malvidin-3-*O*-glucoside. The products are transient and they later evolve to form substances with a greater degree of condensation [149]. Therefore, these reactions do not only contribute to the color change observed during wine aging, but do contribute to the decline in astringency. Though cool aging temperatures can retard the aldehyde mediated polymerization between anthocyanins and flavan-3-ols, it can limit the fast formation of excessively large pigments, their subsequent precipitation, and hence color loss [140,152]. Furthermore, the presence of oxygen and lower pH values can promote such reactions, since the formation of acetaldehyde and its protonated form are favored under such conditions, respectively [121,140,153]. However, previous studies have only focused on the reactions involving smaller proanthocyanidins and the monomeric flavan-3-ols in model solutions, so the relative importance of the bigger polymeric proanthocyanidins is still unclear.

Additionally, besides acetaldehyde, some minor aldehydes in red wine, such as propionaldehyde, isovaleraldehyde, isobutyraldehyde, benzaldehyde, formaldehyde, 2-methybutyraldehyde, vanillin, furfural and hydroxymethylfurfural can also mediate the condensation to add further complexity through the similar mechanism described for acetaldehyde [33,60,142,154,155,156]. For example, the glyceraldehyde and dihydroxyacetone generated from the oxidation of glycerol, which is the second most common alcohol in red wines, can promote the formation of the novel cross-linked anthocyanin based pigments [33]. Propionaldehyde can mediate condensation reaction between malvidin-3-*O*-glucoside and (+)-catechin to lead to the formation of malvidin-3-*O*-glucoside-(C8→)-propyl-(C8→)-(+)-catechin [60]. In the presence of furfural or its derivative 5-hydroxymethylfurfural, the reactions between (+)-catechin and anthocyanins were also detected to produce various furfuryl or 5-hydroxymethylfurfuryl group linked oligomers [154]. 

Also glyoxylic acid bearing an aldehyde moiety can mediate indirect condensation of anthocyanins and flavan-3-ols. Similar to the acetaldehyde-mediated polymerization, glyoxylic acid from iron-catalyzed oxidation of tartaric acid attacks the nucleophilic C8 or C6 positions in the A ring of anthocyanins and flavan-3-ols to produce a carboxy methane bridged oligomers [141]. Considering the relative amounts of the tartaric acid and ferrous ions in wine, this reaction may be quite important during wine aging [157]. It probably competes with the acetaldehyde-mediated polymerization and contributes considerably to color alteration in the aging wine [158]. 

Detailed mass spectrometry information of some major products from aldehyde-mediated condensation reactions of anthocyanins and/or flavan-3-ols that can be detected in aged red wines or be synthesized in model wine solutions is summarized in Table 3, as shown below [63,64,66,128]. Although most of them are synthesized in model solutions, which usually are detected in trace amounts or cannot be detected in most aged red wines, they may also contribute considerably to the wine color evolution and mouth feel of red wines, as well as the diversity of anthocyanin derivative pigments [47,79,138,144,154]. 

**Table 3:** The mass spectral data of some major products from aldehyde-mediated condensation reactions of anthocyanins and/or flavan-3-ols [63,64,66,128].

Compounds	Molecular ion M^+^ (*m/z*)	Fragment ion M^+^ (*m/z*)
Petunidin-glucoside-8-ethyl-(epi)catechin	795	633,505,481,435,328
Malvidin-glucoside-8-ethyl-gallocatechin	825	
Malvidin-glucoside-8-ethyl-(epi)catechin	809	657,517,357,341,331
Malvidin-acetylglucoside-8-ethyl-(epi)catechin	851	
Malvidin-coumaroylglucoside-8-ethyl-(epi)catechin	955	803,665,647,357,341
Peonidin-glucoside-8-ethyl-(epi)catechin	779	
Peonidin-coumaroylglucoside-8-ethyl-(epi)catechin	925	635,617,327
Malvidin-glucoside-4-methyl-(epi)catechin	795	505
Malvidin-glucoside-4-2methylpropyl-(epi)catechin	837	547
Malvidin-glucoside-4-3methylbutyl-(epi)catechin	851	561
Malvidin-glucoside-4-2methylbutyl-(epi)catechin	851	561
Malvidin-glucoside-4-benzyl-(epi)catechin	871	581
Malvidin-glucoside-4-propyl-(epi)catechin	823	533
Malvidin-glucoside-4-ethyl-PC dimer	1097	519
Malvidin-glucoside-4-3methylbutyl-PC dimer	1139	
Malvidin-glucoside-4-benzyl-PC dimer	1159	707
Malvidin-glucoside-4-propyl-PC dimer	1111	959
Malvidin-glucoside-4-ethyl-epicatechin-3-*O*-gallate	961	799,519
Malvidin-glucoside-4-ethyl-B2-3'-*O*-gallate	1249	
Malvidin-glucoside-4-3methylbutyl-epicatechin-3-*O*-gallate	1003	
Malvidin-glucoside-4-3methylbutyl-B2-3'-*O*-gallate	1291	
Malvidin-glucoside-4-benzyl-epicatechin-3-*O*-gallate	1023	719
Malvidin-glucoside-4-benzyl-PB2-3'-*O*-gallate	1311	419,581
Malvidin-glucoside-4-propyl-epicatechin-3-*O*-gallate	975	813,671
Malvidin-glucoside-4-propyl-PB2-3'-*O*-gallate	1263	821
Malvidin-glucoside-4-methyl-epicatechin-3-*O*-gallate	947	785,343
Malvidin-glucoside-4-methyl-PB2-3'-*O*-gallate	1235	1073,793
Malvidin-glucoside-4-isobutyl-epicatechin-3-*O*-gallate	989	385
Malvidin-glucoside-4-isobutyl-PB2-3'-*O*-gallate	1277	835

### 3.3. Xanthylium Pigments

**Figure 7:**
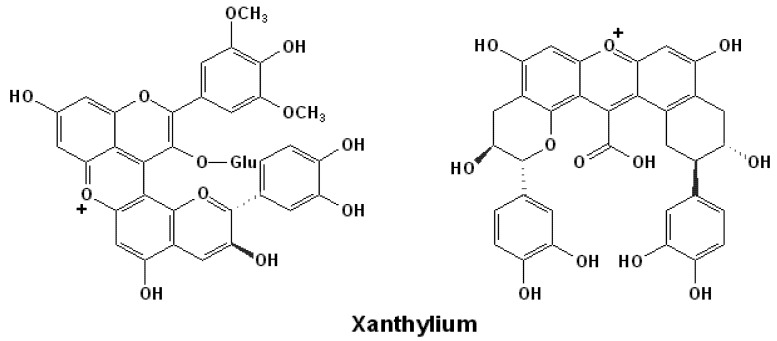
Structures of some xanthylium pigments [159,160,161,162].

Xanthylium salts are one group of important polyphenolic pigments which are considered to be derived by direct or indirect condensation of flavonoids, especially with anthocyanins and/or flavan-3-ols. Such condensed flavonoid oligomers can be converted to corresponding xanthene derivatives by subsequent cyclization, which can be further oxidized to form the yellow to red xanthylium pigments, as shown in Figure 7 [159,160,161,162]. Xanthyliums have various sources in red wines.

The A-T adducts can generate yellow-orange xanthylium pigments by further structural rearrangements. After the dehydration reaction between the hydroxyl groups on the C5 position in the A ring of the anthocyanin moiety and the C8 position in the A ring of the flavan-3-ol unit, a new heterocyclic pyran ring is formed between the two parent rings and the xanthylium structure is generated [108,115,152,163,164]. However, they are also proposed to be formed directly from oligomeric flava-3-ols [157,165].

The glyoxylic acid mediated dimer of flavan-3-ols and anthocyanins can also generate xanthylium pigments by a similar mechanism. For example, carboxymethane bridged anthocyanins and/or (+)-catechin dimers can be converted into a xanthene derivative by a dehydration reaction involving the hydroxyl groups on the C7 positions, and the product can be further oxidized to xanthylium pigments [166,167]. Similarly xanthylium can also be synthesized from formaldehyde or 2-methybutyraldehyde mediated flavonoid dimers, such as (+)-catechin-furfuryl/-hydroxymethylfurfuryl-malvidin-3-*O*-glucoside dimers [154].

## 4. Influence of Aging Practices on Anthocyanins and Their Derivatives in Red Wines

During wine aging, the concentrations of monomeric and copigmented anthocyanins decrease progressively [14,16,24,25,168]. In particular, acylated anthocyanins disappear a little more quickly than the non-acylated ones. It has been suggested that acylated anthocyanins might undergo hydrolysis into their non-acylated forms during aging. But, different non-acylated free anthocyanins almost share the same depleting kinetics, and pyranoanthocyanins show a similar or lower disappearance rate than their corresponding free precursors [16]. While the monomeric anthocyanins decline more, polymeric anthocyanins are formed helping to maintain the red wine color, albeit with a change of hue.

Oxygen is very important for the evolution of anthocyanins in red wines, particularly during wine aging [121]. Whereas strong oxygenation increases wine oxidation, mild oxygenation during storage can improve wine quality by stabilizing wine color [169]. As already discussed, oxygen is involved in a range of mechanisms, such as the formation of direct or indirect polymeric anthocyanin pigments [119,170]. Previous research has been principally focused on wine aging in wood barrels with permeation of oxygen and more recently has been expanded to include aging in stainless steel tanks with controlled oxygenation [170]. 

Micro-oxygenation is one of the techniques used to promote the evolution of anthocyanins in red wines [169,171,172,173,174,175]. It involves the formation of micro-bubbles through the injection of gaseous oxygen into the wine by means of a micro-diffuser, or the controlled diffusion of oxygen through a permeable membrane [173]. Research results indicate that the effect of this technique on wine color can be compared to that of the maturation in oak barrels, especially with the addition of oak chips or staves [172,174,175]. Normally, the application of micro-oxygenation can result in a lower concentration of monomeric anthocyanins and a higher concentration of polymeric anthocyanins and pyranoanthocyanins in red wines [169,171,172,173,174,175]. It can be followed by aging in barrels or bottles to further modify the color properties of the wine. Red wines with lower phenolic content have been found to be less influenced by micro-oxygenation and may even suffer over-oxygenation [169]. Thus, such enological techniques need to be use after careful consideration. 

When aging in oak, besides oxygen permeation, many compounds, such as gallic, ferulic, vanillic, syringic, ellagic acids, ellagitannins and tannins are extracted into wine, thereby introducing desirable oak aromas and flavors into red wines, as well as involving in a number of reactions with wine phenolics, such as anthocyanins and tannins modifying the palate structure of the wine [14,24]. Though aging in oak barrels is traditional and is beneficial, oak chips can be used in wine aging, frequently in conjunction with micro-oxygenation, to reduce costs [172,174,176,177]. In some research, red wines previously in contact with oak chips or staves lost their monomeric anthocyanins more quickly and showed a more rapid increase in polymerization than those aged in barrels. In some cases this resulted in the premature development of red-brown hues [176,177]. Some researchers have suggested that the barrel wood type had a significant influence on the chromatic characteristics of red wines. For example, maturation and aging in chestnut barrels has produced different results to those from oak aging because of their greater quality of oxygen penetrating through chestnut than oak barrels [178]. Some studies showed that the red wines aged with French oak lost slightly more monomeric anthocyanins than those aged in Hungarian or American oak, whereas others have reported that red wine aged in Hungarian oak barrels suffered a slightly higher loss of monomeric anthocyanins than those treated with French and American oak wood [176,177]. This perhaps is not surprising given the natural variation in wood composition combined with differences in seasoning and coopering by the different barrel makers. So perhaps it is not surprising that some studies have also suggested that the type of container, an oak barrel or a stainless-steel tank, has little influence on the anthocyanin fingerprints in red wines [170].

After fermentation the lees are not always removed immediately and some wines are aged with lees for 3–8 months, the technique of *bâtonnage* [179]. During this time, though the yeast cell walls could well adsorb some anthocyanins, the dead yeast cells may also release some compounds that could contribute to wine aging by improving wine stabilization in terms of color and proteins [179,180]. Some studies also revealed that aging over lees appears to have a protective effect on the total monomeric anthocyanin content [172,179,180]. Besides, as mentioned above, pyruvic acid and acetaldehyde can be involved in the formation of pyranoanthocyanins, and acetaldehyde also contribute to the synthesis of bridged anthocyanin pigments.

During aged with lees, some polysaccharide can be released from the cell wall after yeast autolysis [181]. As one of the major polysaccharide groups present in wine, mannoproteins are highly glycosylated and play a series of important roles in wines, such as to adsorb ochratoxin A, to enhance malolactic bacteria growth, to inhibit tartaric salts crystallization, to prevent protein haziness, to enhance and interact with some wine aromas, as well as to react with anthocyanins, tannins or other phenolics in wines [181,182,183,184,185,186,187,188]. In recent years some researches aimed to evaluate the influence of mannoproteins in the color stability in red wines were conducted by using commercial mannoproteins or mannoprotein overproducing yeast strain. The results usually suggested that there was no inﬂuence on color stability but these mannoproteins can delay the tannin polymerization in red wines [189,190,191,192]. Considering the crucial role of tannins in the formation of polymeric anthocyanin pigments, they may take effect in the long-term stability of the wine color.

## 5. Conclusions

**Scheme 11:**
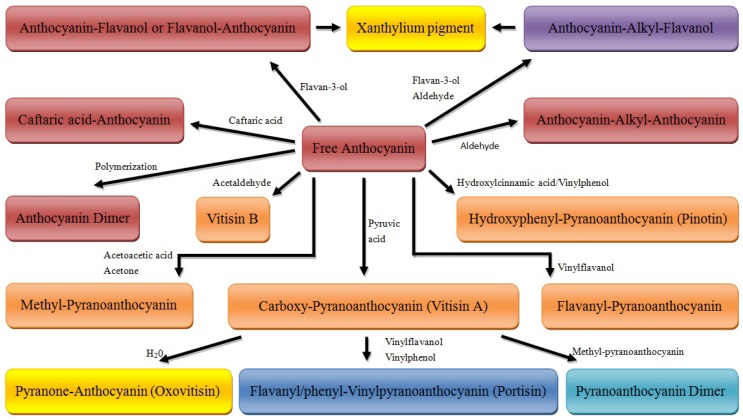
Evolution of anthocyanins in aged red wines [14,24,57].

During winemaking and wine aging, anthocyanins in red wines undergo complicated chemical changes to form diverse anthocyanin derivatives, as summarized in Scheme 11 [14,24,57]. Accurate knowledge of their variation is crucial for better understanding and control of color evolution. Numerous by-products of yeast metabolism not only play a role in copigmentation, which is the first step in the formation of stable pigments in red wines, but also in the further formation of the new pigments, such as the pyranoanthocyanins and the polymeric anthocyanin pigments [193]. Although some of these pigments have been detected in only very small quantities in red wines, they have unique spectroscopic features and together may contribute noticeably to the overall hue and stability of color of aged red wines. Thus, factors such as yeast species and strains, temperature, pH, and the concentration of sulfur dioxide during fermentation, as well as various enological practices will all influence the profiles of anthocyanin and their derivatives, leading to diverse color expression.

The areas for future research into anthocyanin derivatives, their chemistry and behavior during red wine maturation and aging is still expanding and there is much to be done. Some of the fields requiring extensive investigation include:

(1) Identification of new pyranoanthocyanins in aged red wines, especially the new pigments from second generation of carboxy- or methyl-pyranoanthocyanins.
(2) Identification of more complicated polymeric anthocyanins in aged red wines, especially the ones with higher polymerization degree and new configurations.
(3) Quantification of the amounts of such pigments and their extinction coefficients to enable the assessment of their contribution to visual wine color.
(4) New enology practices to improve the production and stability of anthocyanin derivatives, as well as total wine color.
